# Spectrofluorimetric determination of selected genotoxic impurities in pharmaceutical raw materials and final products

**DOI:** 10.1038/s41598-022-19603-9

**Published:** 2022-09-12

**Authors:** Aliaa I. Shallan, Ali Abdel-Hakim, Mohamed A. Hammad, Maha M. Abou El-Alamin

**Affiliations:** 1 Department of Pharmaceutical Analytical Chemistry, Faculty of Pharmacy, Helwan University, Cairo, 11795 Egypt; 2 Department of Analytical Chemistry, Faculty of Pharmacy, University of Sadat City, Sadat City, 32897 Egypt

**Keywords:** Analytical chemistry, Photochemistry

## Abstract

A green spectrofluorimetric method was introduced for the determination of selected genotoxic impurities; 2-aminopyridine and 3-aminopyridine in different pharmaceutical raw materials and dosage forms. The method relied on the native fluorescence of these impurities in acidic medium. The experimental conditions were carefully studied and optimized, and the method was validated according to International Council on Harmonisation (ICH) guidelines. The linear range for both analytes was 2.50–100 ng/mL with good determination coefficients of 0.9995 and 0.9992 and detection limits of 0.62 ng/mL and 0.74 ng/mL for 2-aminopyridine and 3-aminopyridine, respectively. The method was successfully applied for determination of 2-aminopyridine and 3-aminopyridine in four active pharmaceutical ingredients and nine dosage forms with satisfactory percentage recoveries and without interference from co-formulated excipients. Analytical performance of the proposed method was comparable to that of the reported methods; hence, the proposed method can be used as a simple and low-cost alternative in quality control laboratories.

## Introduction

Impurities are often present in synthetic active pharmaceutical ingredients (APIs) as part of the process, originate from different sources such as starting materials, intermediates, by-products, catalysts, reagents, and solvents. Impurities can be genotoxic and may cause deleterious effects on human body through destructive effects on the DNA and chromosomes^1,2^. Genotoxic impurities (GTIs) may be organic impurities such as alkyl halides^3^, or inorganic (elemental) impurities such as cadmium, lead and chromium^4–6^. Different regulatory agencies set strict limits on the presence of impurities in pharmaceutical raw materials and dosage forms^3^.

**Figure 1:**
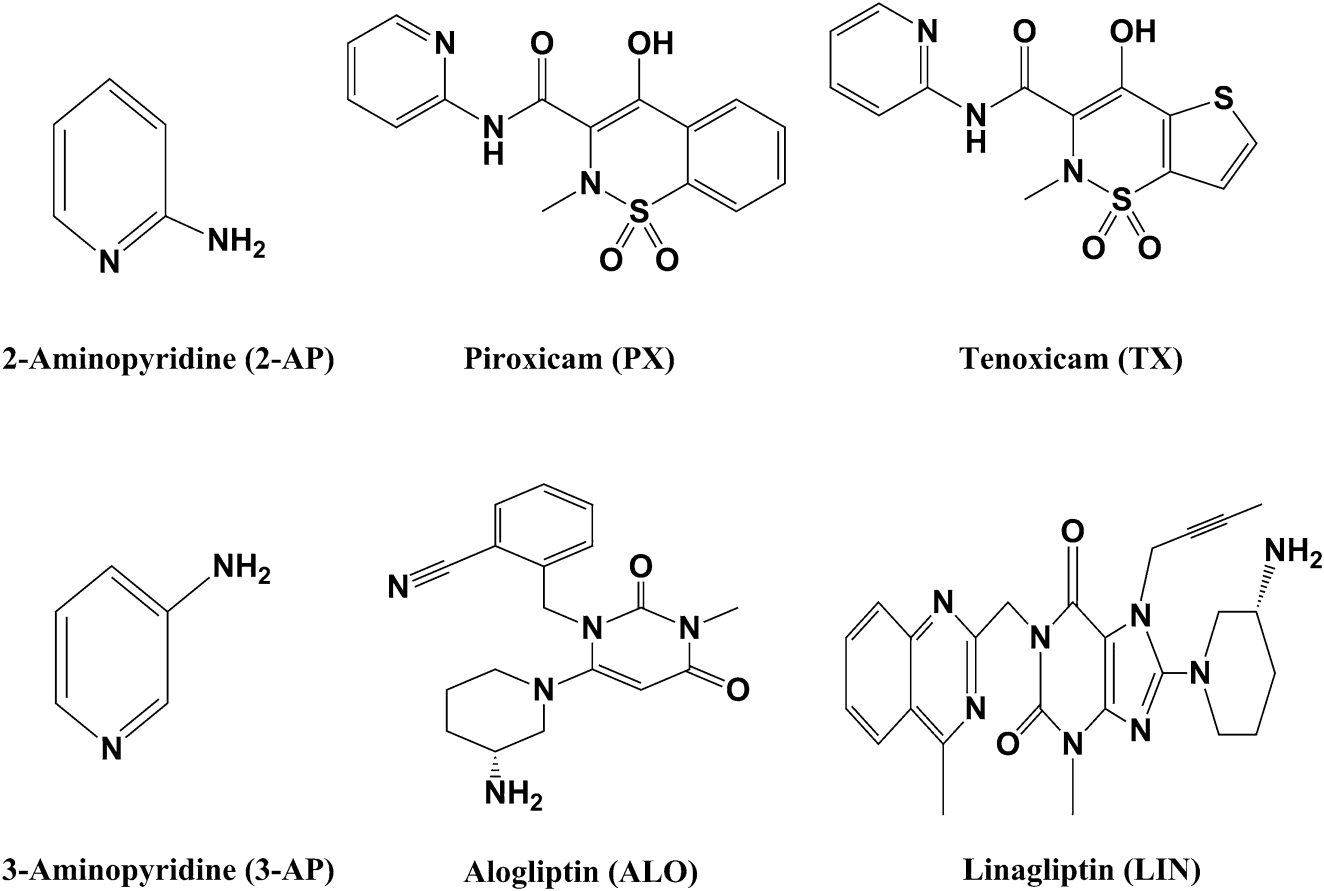
Analytes and drug compounds structures.

Pyridine derivatives are one class of these strictly regulated impurities, 2-aminopyridine (2-AP) and 3-aminopyridine (3-AP) (Fig. 1) are considered as potential GTIs^7^. Pyridine and its derivatives are commonly used in the manufacturing of pharmaceuticals, pesticides, rubbers and dyes as starting material or intermediate^8^. Table 1 lists some of the pharmaceutical APIs and respective dosage forms in which these impurities must be monitored and controlled.

**Table 1:** Reported analytical methods for determination of 2-AP and 3-AP.

Impurity	APIs	Dosage forms	Technique	Linear range (µg/mL)	LOD (µg/mL)	References
2-AP	PX	Capsules Suppository	Derivative spectrophotometry	4.7–47	–	^18^
	PX and TX	PX tablet	Spectrofluorimetry	0.001–0.02	0.16 × 10^–3^	^16^
		PX ampoule				
		TX tablet				
		TX vial				
	PX	–	HPLC	2–40	–	^19^
	TX	Tablet	Derivative spectrophotometry	0.3–2	–	^20^
			HPLC	0.3–2	–	
3-AP	Dalfampridine	–	HILIC-UV^a^	22.5–112.5	7.44	^1^
	LIN	–	HILIC-UV^a^	30.0–450.0	7.5	^13^
	Typical pharmaceutical compounds	Vitamin C	LC–MS^b^	1–50	0.05	^21^
		Bromhexine				
		Ephedrine				
		Penicillin V				
2-AP and 3-AP	PX, TX, ALO and LIN	PX tablet	Spectrofluorimetry	2.50 × 10^–3^–100 × 10^–3^	0.62 × 10^–3^ for 2-AP 0.74 × 10^–3^ for 3-AP	This work
		PX capsule				
		PX ampoule				
		PX gel				
		PX suppository				
		TX tablet				
		TX capsule				
		TX vial				
		TX suppository				

^a^Hydrophilic interaction liquid chromatography.

^b^Liquid chromatography-mass spectrometry.

Piroxicam (PX) and tenoxicam (TX) (Fig. 1) are long acting non-steroidal anti-inflammatory drugs (NSAIDs), characterizing by their powerful anti-inflammatory and analgesic effects, both of them are effectively used in the management of bone and joint diseases and pain related disorders^9^. The British Pharmacopeia (BP) lists 2-AP as a potential impurity in both PX and TX^10^.

Alogliptin (ALO) and linagliptin (LIN) (Fig. 1) are effective oral anti-diabetic agents belonging to a family known as dipeptidyl peptitase-4 (DPP-4) inhibitors, administered once daily for patients with type-2 diabetes mellitus, with maximum daily doses of 5 mg and 25 mg of ALO and LIN, respectively^11,12^. 3-AP is an intermediate in the synthesis of ALO and LIN raw materials^13–15^. Therefore, it may present in ALO and LIN final drug substance.

According to BP, the amount of 2-AP in PX and TX should not exceed 0.2% in bulk drug and 0.25% in pharmaceutical preparations, the assay is based on HPLC with UV detection^10,16^. Also, 3-AP must be controlled in ALO and LIN drug substances according to guidelines for GTIs. According to International Council on Harmonization (ICH) M7 guideline, the maximum allowed limit of single GTI in API is 1.5 µg/day^17^. Based on the above mentioned maximum daily doses of ALO and LIN, the estimated permissible quantities of 3-AP in ALO and LIN are 60 ppm and 300 ppm, respectively.

Literature review revealed few methods based on optical or chromatographic techniques were reported for determination of 2-AP and 3-AP. These methods are summarized in Table 1.

As shown in Table 1, the previously reported methods either lack adequate sensitivity or require sophisticated instruments or applied only in limited number of dosage forms, therefore, development of sensitive and simple method for determination of 2-AP and 3-AP in raw materials and different dosage form is of great importance.

The aim of this work is to develop and validate a highly sensitive, simple, and economic spectrofluorimetric method for the determination of 2-AP in PX and TX and the determination of 3-AP in ALO and LIN. The high sensitivity of the proposed method allows the determination of 2-AP and 3-AP below their permissible limits in APIs and different dosage forms.

The current spectrofluorimetric approach is superior to previously published methods in its low-cost, simplicity and availability compared to chromatographic methods, and its high sensitivity compared to UV-spectrophotometric methods. Moreover, the published spectrofluorimetric method for determination of 2-AP was applied only in APIs, tablets, and injectable preparations. However, our suggested method is applied for determination of 2-AP in APIs, tablets, capsules, injectable preparations, gel, and suppositories dosage forms.

## Experimental

### Apparatus

Fluorimetric measurements were done using Jasco model FP-8300 (Tokyo, Japan) spectrofluorimeter equipped with 1-cm quartz cuvette. The excitation and emission slit widths were adjusted at 5 nm. Spectra Manager software (Jasco Co., Tokyo, Japan) was applied for data processing and acquisition.

pH was measured and adjusted using pH-meter, Jenway 3510 (UK).

Thermostatically controlled water-bath, SY-2L4H (China), benchtop centrifuge, Cyan-CL008 (Belgium) and ultrasonic cleaner, SB-120DT (China) were also used.

### Materials

#### Reference samples

2-AP (99.0%) and 3-AP (99.0%) were purchased from Sigma Aldrich (St. Louis, USA).

PX of purity (99.7%) was kindly supplied by EL-Obour Modern Pharmaceutical Industries Company (El-Obour City, Egypt), TX of purity (100.1%) was kindly supplied by EIPICO Company (Cairo, Egypt), ALO of purity (99.7%) was kindly supplied by Global Napi Pharmaceutical Company (6th of October, Egypt), LIN of purity (99.8%) was kindly supplied by EVA Pharma Company (Cairo, Egypt).

#### Pharmaceutical preparations

Different dosage forms were purchased from the local market. The composition of each were as follow:

Brexin tablets (product of Chiesi Pharma Company): labelled to contain (20 mg PX/tablet).

Feldene capsules, ampoules, and gel, (products of Pfizer Pharmaceutical Company) labelled to contain (20 mg PX/capsule and ampoule, 0.5% PX gel).

Dispercam suppositories (product of Medical Union Pharmaceuticals): labelled to contain (20 mg PX/suppository).

Epicotil tablets, vials, and suppositories, (products of EIPICO Company) labelled to contain (20 mg TX/tablet, vial and suppository).

Soral capsules (product of Global Napi Pharmaceutical Company) labelled to contain (20 mg TX/capsule).

#### Chemicals

Sulphuric acid (H_2_SO_4_) (98%) was obtained from Piochem (Cairo, Egypt).

Sodium dihydrogen phosphate, phosphoric acid, methanol, ethanol and acetonitrile were purchased from Merck (Darmstadt, Germany).

Sodium dodecyl sulfate (SDS), Tween 20, Tween 80 and span 60 were purchased from Sigma Aldrich (St. Louis, USA).

Deionized water was used during the whole experiments.

### Preparation of stock solutions and buffer solution

Stock standard solution equivalent to 100 µg/mL of each of 2-AP and 3-AP was prepared separately by transferring accurately weighed 10.0 mg of 2-AP and 3-AP into 100-mL volumetric flask and completed to the mark with deionized water. Additional dilution was made with the same solvent to get working standard solutions with concentration of 1.00 µg/mL.

100 mM phosphate buffer (pH 3.0) was prepared by dissolving 12.0 g of sodium dihydrogen phosphate in 800-mL deionized water, the pH was adjusted by phosphoric acid, then diluted to 1000-mL using the same solvent^22^.

### Construction of calibration curves

*For 2-AP,* into a series of 10-mL volumetric flasks, appropriate aliquots from working standard solution covering concentration range of 2.50–100 ng/mL were added and mixed with 1.0 mL of 100 mM phosphate buffer of pH 3.0 (to get final buffer concentration of 10 mM), then, completed to the mark with deionized water to obtain final concentration range of 2.50–100 ng/mL.

*For 3-AP,* the same procedure was followed, except that 100 mM H_2_SO_4_ was used instead of phosphate buffer.

Fluorescence intensity was recorded at (λ_em_370 nm/λ_ex_298 nm) for 2-AP and (λ_em_403 nm/λ_ex_250 nm) for 3-AP. The calibration curves were obtained by plotting fluorescence intensity versus concentration of analyte (ng/mL) and the corresponding regression equations were derived.

### Assay procedures for active pharmaceutical ingredients

Accurately weighed 10.0 mg of PX and TX and 100.0 mg of LIN and ALO were separately transferred into a series of 100-mL volumetric flasks. Spiked with appropriate aliquots of 2-AP (for PX and TX) and 3-AP (for ALO and LIN) to obtain final 2-AP and 3-AP concentrations of 2.50–100 ng/mL. The mixture was dissolved in 10-mL methanol then sonicated for 5 min, the volume was completed to the mark with deionized water, then further diluted with deionized water to obtain a final concentration of 10 μg/mL of PX and TX, 100 μg/mL of ALO and LIN. Then, the procedure described under *Construction of calibration curve* was performed.

### Assay procedures for dosage forms

All dosage forms were spiked with appropriate aliquots of 2-AP and 3-AP working standard solutions to obtain final concentrations of 2.50–100 ng/mL. The final concentration of both PX and TX of each dosage form was adjusted to be 10 μg/mL. Then, the procedure described under *Construction of calibration curve* was performed.

#### Procedures for tablets

Ten Brexin tablets and Epicotil tablets were finely powdered separately and thoroughly mixed. An accurately weighed amount equivalent to 10 mg of PX and TX were transferred separately into a series of 100-mL volumetric flasks and dissolved in 10-mL methanol, and spiked with increasing concentrations of 2-AP, the flasks were sonicated for 5 min and completed to the volume with deionized water. The obtained solutions were filtered and further diluted with deionized water.

#### Procedures for capsules

The drug content of ten Feldene capsules and Soral capsules were separately emptied, weighed, mixed. An accurately weighed amount equivalent to 10 mg of PX and TX were transferred separately into a series of 100-mL volumetric flasks and the procedure was completed as mentioned under Tablets.

#### Procedures for ampoules and vials

The content of ten Feldene ampoules were mixed and a volume equivalent to 20 mg of PX was transferred into a series of 100-mL volumetric flasks, then, spiked with increasing concentrations of 2-AP and completed to the volume with deionized water. The obtained solutions were further diluted with deionized water.

Ten Epicotil vials were reconstituted with deionized water, thoroughly mixed, and transferred into a series of 100-mL volumetric flasks, the vials were washed with deionized water and the washing was transferred into the same flasks and spiked with increasing concentrations of 2-AP, then, completed to the volume with deionized water. The obtained solutions were further diluted with deionized water.

#### Procedures for gel

An amount of Feldene gel equivalent to 10 mg of PX was weighed in a beaker, spiked with increasing concentrations of 2-AP and allowed to dissolve in 25 mL ethanol and 2.0 mL NaOH solution, after that, the solutions were filtered and transferred into a series of 100-mL volumetric flasks and completed to the volume with deionized water. The obtained solutions were further diluted with deionized water.

#### Procedures for suppositories

An accurately weighed amounts of Dispercam suppositories and Epicotil suppositories equivalent to 10 mg of PX and TX were separately placed in a beaker, spiked with increasing concentrations of 2-AP and melted in a thermostatically controlled water-bath adjusted to 50–60 °C, then, 5.0 mL methanol was added and then heated again with gentle shaking, then, the solutions were transferred into a series of 100-mL volumetric flasks and completed to the volume with deionized water, the solutions were then centrifuged, filtered and further diluted with deionized water.

## Results and discussion

2-AP and 3-AP are efficient fluorophores as a result of (π-π^*^) nature of the lowest excited state^23^. Meanwhile, the cited drugs (PX, TX, ALO and LIN) are not or very weakly fluorescent compounds^24–27^, therefore, 2-AP and 3-AP can be determined quantitatively without any interference from these drugs. Different parameters that affect the fluorescent signals of 2-AP and 3-AP were thoroughly studied and optimized.

### Fluorescence spectrum

**Figure 2:**
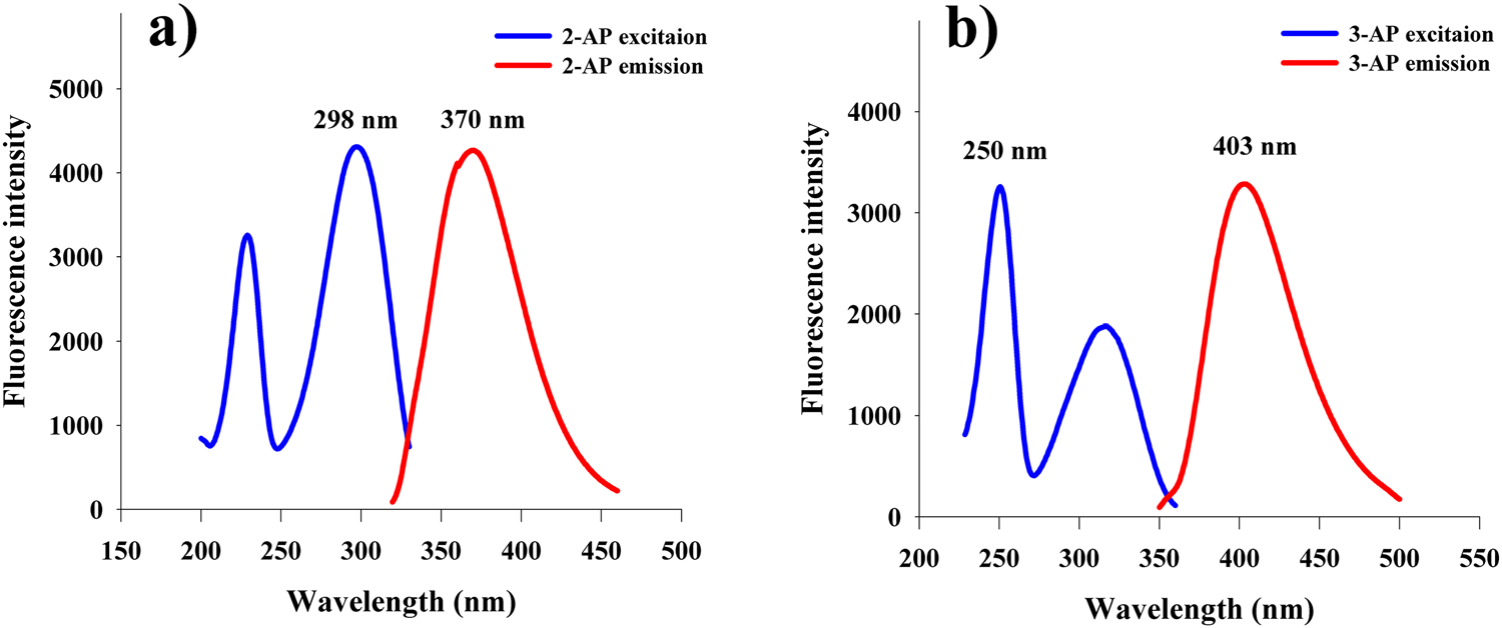
Excitation and emission spectra of 50.0 ng/mL of (**a**) 2-AP in 10 mM phosphate buffer of pH 3.0 and (**b**) 3-AP in 10 mM H_2_SO_4_.

The native fluorescence spectra of 2-AP and 3-AP in acidic media were recorded. 2-AP and 3-AP exhibited strong native fluorescence at 370 nm and 403 nm after excitation at 298 nm and 250 nm for 2-AP and 3-AP, respectively, as presented in Fig. 2.

### Experimental parameters optimization

#### Selection of optimum pH

**Figure 3:**
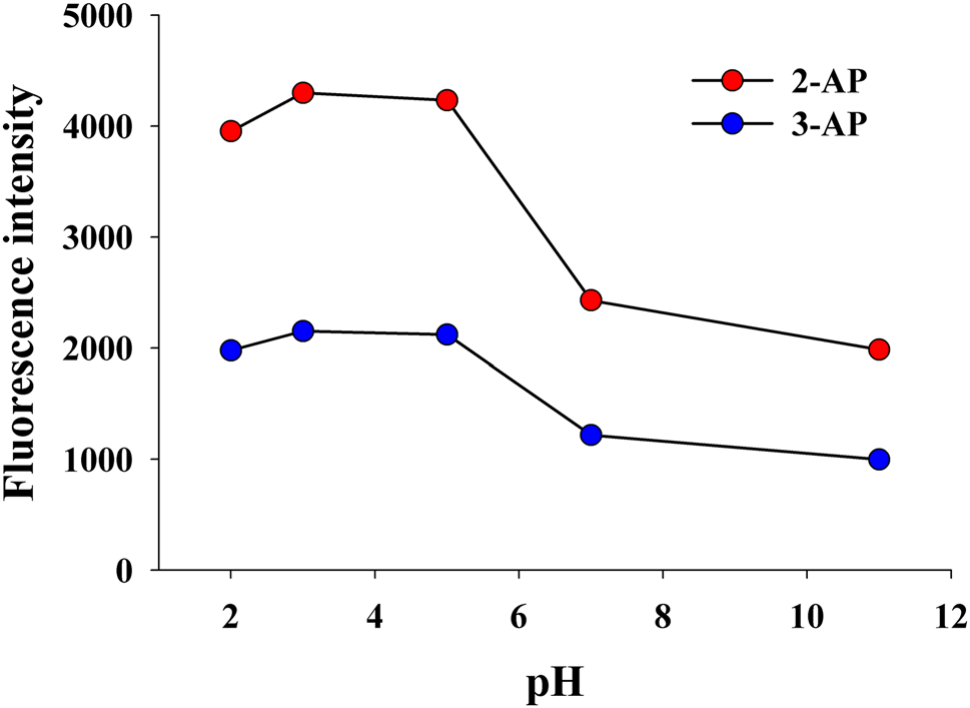
Effect of pH of 10 mM B-R buffer on the fluorescence intensities of 2-AP and 3-AP (50.0 ng/mL).

An initial assessment was performed to study the influence of pH on the fluorescence intensities of 2-AP and 3-AP, as pH value affects the ionization of the studied aminopyridines. The effect of pH was studied using 10 mM Britton-Robinson (B-R) buffer through pH range of 2.0–11.0 as shown in Fig. 3. The results clearly showed that, the fluorescence intensities of 2-AP and 3-AP were found to be pH dependent, the fluorescence intensities sharply quenched in neutral and alkaline media. The highest fluorescence intensities for both, 2-AP and 3-AP were obtained at pH 3.0. The obtained results were in good agreement with those obtained by previously published results^23^.

#### Effect of type and concentration of acidic solutions

In this study, the fluorescence intensities of 2-AP and 3-AP in different acidic solutions were examined using different buffers, namely, acetate, citrate, glycine, phosphate and B-R buffers with pH value of 3.0, H_2_SO_4_ was also included, while HCl was excluded in this study because of its quenching effect on the native fluorescence of 2-AP and 3-AP as a result of external heavy atom effect of the halide ion^28^.

**Figure 4:**
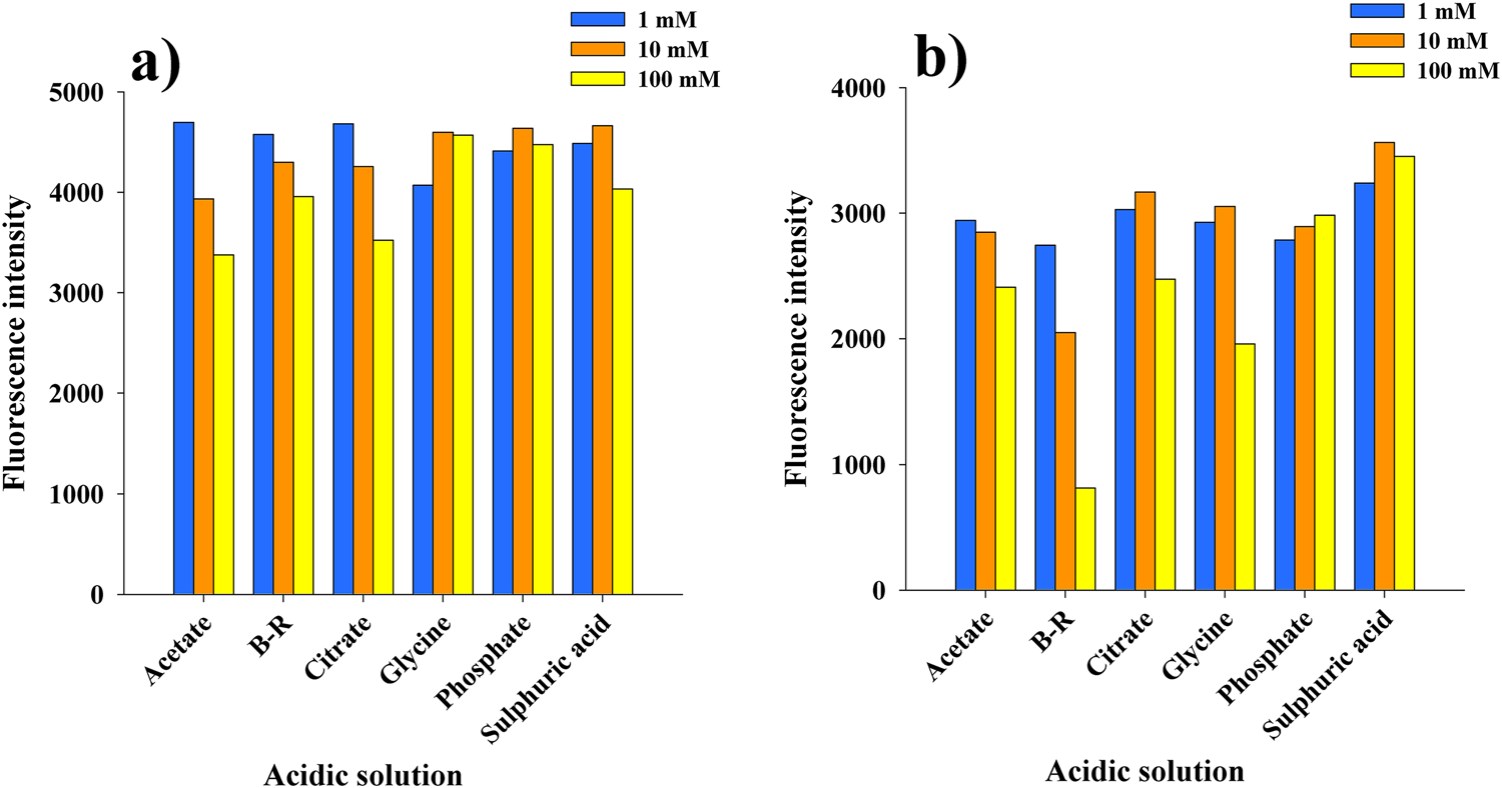
Effect of different acidic solutions with different ionic strengths on the fluorescence intensities of 50 ng/mL of (**a**) 2-AP and (**b**) 3-AP.

Different concentrations (100 mM, 10 mM and 1 mM) for each acidic solution were also compared as shown in Fig. 4. The results indicated that, the fluorescence intensity of 2-AP in phosphate buffer was not significantly affected by its ionic strength compared to other studied acidic solutions, therefore, 10 mM phosphate buffer was selected as the optimum buffer. While for 3-AP, 10 mM of H_2_SO_4_ was selected as the optimum acidic solution because it produced the highest fluorescence intensity.

#### Effect of surfactant

Seeking to beneficial enhancement effect of the micellar media on the fluorescence intensity^29^. The fluorescence behavior of 2-AP and 3-AP in different organized media was examined using different micellar media, namely, SDS, Tween 20, Tween 80 and Span-60 in concentrations above their critical micelle concentrations. 1.0 mL of each surfactant (1.0% w/v) was added to acidic solution of 2-AP and 3-AP, then, the fluorescence intensities were measured.

The obtained results (Supplementary Fig. S1) indicated that, no significant enhancement effect on the fluorescence intensities of 2-AP and 3-AP was observed upon addition of surface-active agent. The method was therefore conducted without the use of any surfactant.

#### Effect of diluting solvent

The influence of the commonly used organic solvents (methanol, ethanol and acetonitrile) on the fluorescence intensities of 2-AP and 3-AP was investigated. Different binary solvent mixtures were studied as shown in Supplementary Fig. S2. The results indicated that, up to 40% of the studied organic solvents did not significantly affect the fluorescence intensities of 2-AP and 3-AP. The fluorescence intensities of both 2-AP and 3-AP started to decrease upon addition of more than 40% the studied organic solvents. As a result, water was the diluting solvent during the study.

#### Effect of temperature and fluorescence lifetime

The effect of temperature on the fluorescence intensities of 2-AP and 3-AP was also studied over a temperature ranged from 40 to 100 °C using a thermostatically controlled water-bath. The results (Supplementary Fig. S3) showed that, at high temperatures, the fluorescence intensities of both 2-AP and 3-AP were decreased. This effect might be attributed to internal conversion at higher temperature, accelerated non-radiative deactivation of the excited singlet state^30^, therefore, the whole experiments were performed at room temperature.

The effect of time on the fluorescence intensities of 2-AP and 3-AP was also investigated, it was observed that the fluorescence intensities stayed unaffected for more than 6 h.

### Method validation

The suggested assay was validated in accordance with ICHQ2(R1) guidelines^31^.

#### Linearity range

The linearity of the developed method was assessed by recording the fluorescence intensities of a series of concentrations of each of 2-AP and 3-AP in the range of (2.50–100 ng/mL), calibration plots were constructed by plotting the fluorescence intensities on the y-axis versus concentrations on the x-axis, then, the regression equations were generated by least square treatment of the calibration data. Linear regression analysis of the data gave the following equations:

$$FI = \, 95.386C \, - \, 18.883 \, \left( {r^{2} = 0.9995} \right) \, \;for \, \;2-AP$$

$$FI = \, 77.191C \, + \, 236.16 \, \left( {r^{2} = 0.9992} \right) \, \;for\; \, 3-AP$$

where: *FI* is the fluorescence intensity, *C* is the concentration of analyte (ng/mL), and *r*^2^ is the determination coefficient.

The analytical parameters of the calibration curves are illustrated in Table 2.

**Table 2:** Validation parameters for the determination of 2-AP and 3-AP using the proposed method.

Parameters	2-AP	3-AP
Linear range (ng/mL)	2.50–100	2.50–100
r^2^	0.9995	0.9992
Slope	95.38	77.19
Intercept	− 18.88	236.1
S.D of intercept	17.79	17.36
LOQ (ng/mL)	1.87	2.25
LOD (ng/mL)	0.62	0.74

#### Quantitation limit (LOQ) and detection limit (LOD)

Quantitation and detection limits for 2-AP and 3-AP were calculated through the equations: LOQ = 10* standard deviation of intercept/slope, while LOD = 3.3* standard deviation of intercept/slope (Table 2). The obtained limits indicated high sensitivity of the proposed method for determination of 2-AP and 3-AP in trace amounts.

#### Accuracy and precision

The accuracy and precision of the proposed assay were investigated through 3 concentrations of each of 2-AP and 3-AP within the linear range in 3 replicate manner. The calculated percentage recoveries indicated high accuracy of the proposed method. The analysis was carried out within the same day to evaluate intra-day precision and at 3 successive days for inter-day precision. The obtained low values of %RSD proved the good precision of the proposed method. The results of accuracy and precision are shown in Table 3.

**Table 3:** Evaluation of accuracy and precision of the proposed analytical method.

Analyte	Added concentration ng/mL	Intra-day			Inter-day		
		%Recovery^a^	Mean %Recovery	%RSD	%Recovery^a^	Mean %Recovery	%RSD
2-AP	20.0	98.3	99.4	0.99	98.9	99.4	0.57
	40.0	100			99.2		
	60.0	100			100		
3-AP	20.0	97.7	98.9	1.16	98.9	99.5	0.57
	40.0	98.9			100		
	60.0	100			99.7		

^a^Each result is the average of 3 different determinations.

For further confirmation of the accuracy, the results of the proposed method were statistically compared with those obtained by the reported methods for analysis of 2-AP and 3-AP using Student’s *t-*test and the variance ratio *F*- test. Table 4 revealed that, no significant differences between the proposed method and the reported methods.

**Table 4:** Statistical comparison of the results obtained by the proposed method and the reported methods for determination of 2-AP and 3-AP in pure form.

	2-AP		3-AP	
	Proposed method	Reported method^16^	Proposed method	Reported method^13^
%Recovery^a^	99.3	101	101	99.9
SD	1.13	1.75	0.82	0.42
N	3	3	3	3
*t*-test* (2.77)	0.23		0.25	
*F*-test* (19.00)	2.40		3.81	

*Numbers in parentheses are the tabulated *t* and *F* values at *p* = 0.05.

^a^Each result is the average of 3 different determinations.

### Determination of 2-AP and 3-AP in APIs and dosage forms

The developed method was successfully applied for the determination of 3-AP in ALO and LIN drug substances (Supplementary Table S1) and 2-AP in PX and TX drug substances, in addition, the content of 2-AP was determined in 5 different PX pharmaceutical preparations and 4 different TX pharmaceutical preparations without interference from co-formulated excipients (Supplementary Table S2).

The standard addition technique was applied to compensate for the matrix effect, 3 concentration levels (40.0, 50.0 and 60.0 ng/mL) were spiked in each sample and each experiment was done in triplicate (Supplementary Fig. S4–6). The % recoveries were in the range 96.7–104 with precision (%RSD) lower than 4.60% and r^2^ values at least 0.9973.

The amount of 3-AP and 2-AP in all tested APIs and pharmaceutical preparations was below the allowed limits, but the highest concentrations were found in PX tablet formulation (20.7 ng/mL) and PX suppositories (11.3 ng/mL).

## Conclusion

A fast, economic, simple, and green spectrofluorimetric approach was developed and validated for the determination of two potential genotoxic impurities; 2-AP and 3-AP based on measurement of their native fluorescence in 10 mM phosphate buffer (pH 3.0) at (λ_ex_/λ_em_ of 298/370 nm) for 2-AP and in 10 mM H_2_SO_4_ at (λ_ex_/λ_em_ of 250/403 nm) for 3-AP. The proposed method was validated as per ICH guidelines, the linear range for both impurities was 2.50–100 ng/mL with detection limits of 0.62 and 0.74 ng/mL for 2-AP and 3-AP, respectively, also, the obtained results proved good accuracy and precision. The method was successfully applied for the determination of 2-AP and 3-AP in APIs and different dosage forms below the allowed limits with high percentage recoveries (96.7–104) and low values of %RSD (less than 4.60%), and without interference from co-existing excipients. The proposed method has advantages of being highly sensitive, does not require sophisticated instruments, and capable of analyzing large number of samples in a short period of time with minimal waste. Despite the high toxicity of the 2-AP and 3-AP, exposure can be easily eliminated by using laboratory personal protective equipment. The proposed method can be easily applied in quality control laboratories.

## Supplementary Information

Supplementary Information.

## Data Availability

All data generated or analysed during this study are included in this published article and its Supplementary Information files.
