# Supplementary material for: Spectrofluorimetric determination of selected genotoxic impurities in pharmaceutical raw materials and final products
Source: Sci Rep. 2022 Sep 12;12:15319. doi: 10.1038/s41598-022-19603-9 (PMC9467988; doi:10.1038/s41598-022-19603-9)
Supplement: Supplementary file 1 — Supplementary Information. [file 41598_2022_19603_MOESM1_ESM.doc]

Spectrofluorimetric determination of selected genotoxic impurities in pharmaceutical raw materials and final products

**Aliaa I. Shallan^1^, Ali Abdel-Hakim^*2^, Mohamed A. Hammad^2^, Maha M. Abou El-Alamin^*1^**

^1^Department of Pharmaceutical Analytical Chemistry, Faculty of Pharmacy, Helwan University, 11795 Cairo, Egypt

^2^Department of Analytical Chemistry, Faculty of Pharmacy, University of Sadat City, 32897 Sadat City, Egypt

**^*^**Corresponding authors, e-mail: ali.hassan@fop.usc.edu.eg (Ali Abdel-Hakim),

maha_abdelrehim@pharm.helwan.edu.eg (Maha M. Abou El-Alamin)

**Supplementary Material**


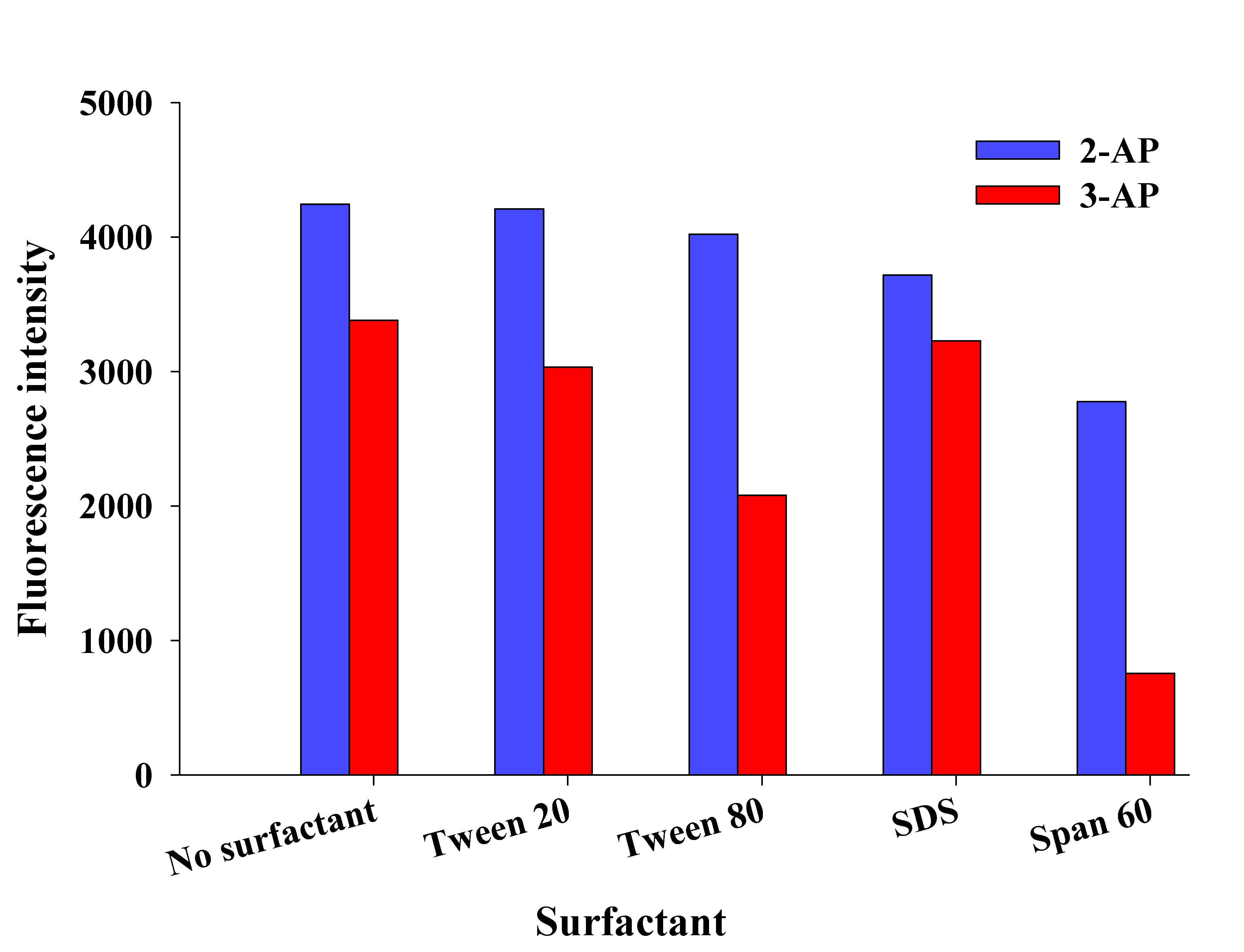


**Figure S1.** Effect of different types of surfactants (1.0 mL of 1.0% solution of each) on the fluorescence intensities of 2-AP and 3-AP (50.0 ng/mL).


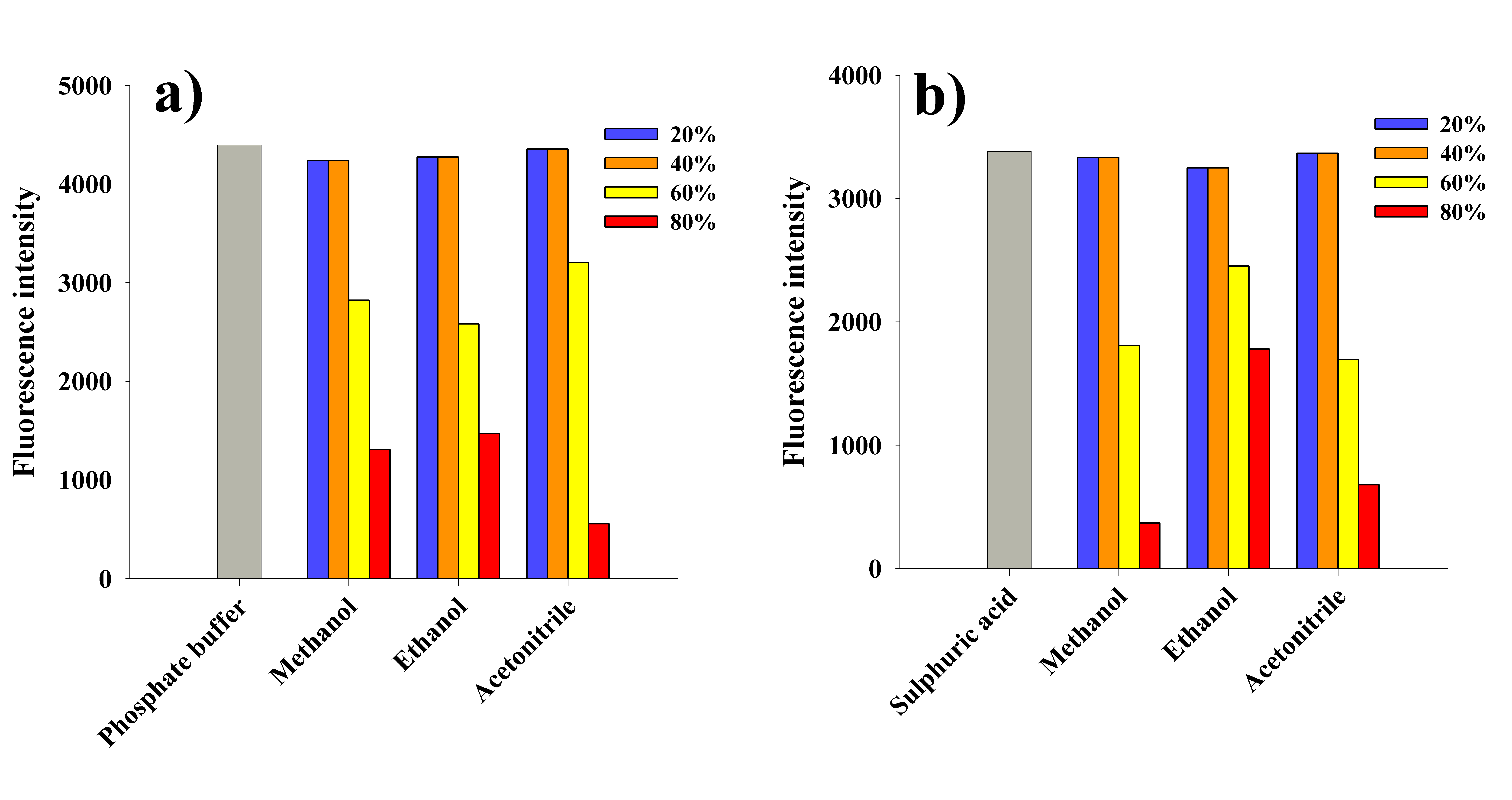


**Figure S2.** Effect of different organic solvents on the fluorescence intensities of 50.0 ng/mL of **(a)** 2-AP and **(b)** 3-AP.


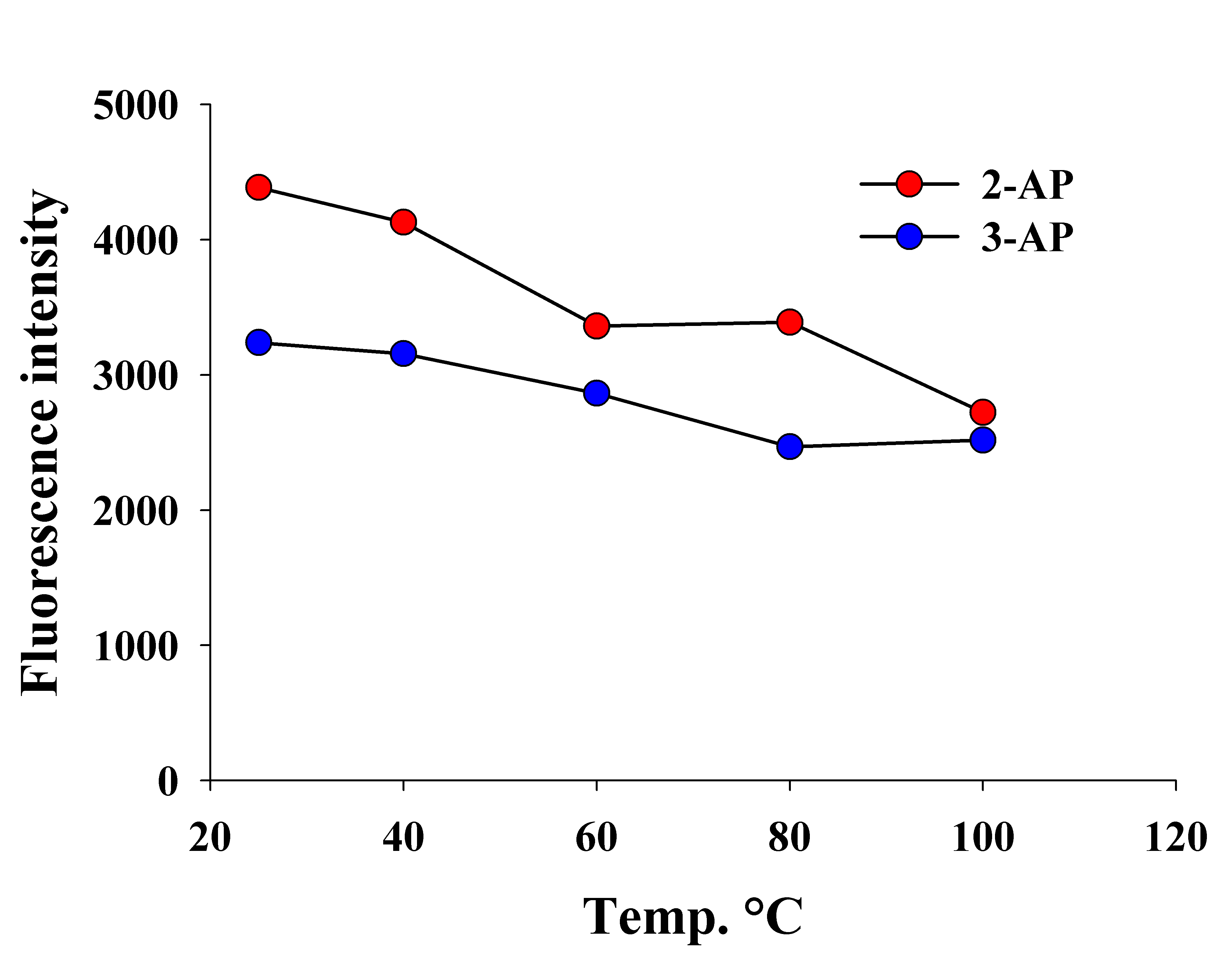


**Figure S3.** Effect of temperature on the fluorescence intensities of 2-AP and 3-AP (50.0 ng/mL).

**Figure S4.** Fluorescence emission spectra under optimal conditions upon addition of different concentrations of 3-AP in **(a)** ALO API and **(b)** LIN API. (From bottom to top: 0 ng/mL, 40.0 ng/mL, 50.0 ng/mL, 60.0 ng/mL).

**Figure S5.** Fluorescence emission spectra under optimal conditions upon addition of different concentrations of 2-AP in **(a)** PX API, **(b)** Brexin tablet, **(c)** Feldene capsule, **(d)** Feldene ampoule, **(e)** Dispercam suppository and **(f)** Feldene gel. (From bottom to top: 0 ng/mL, 40.0 ng/mL, 50.0 ng/mL, 60.0 ng/mL).

**Figure S6.** Fluorescence emission spectra under optimal conditions upon addition of different concentrations of 2-AP in **(a)** TX API, **(b)** Epicotil tablet, **(c)** Soral capsule, **(d)** Epicotil vial and **(e)** Epicotil suppository. (From bottom to top: 0 ng/mL, 40.0 ng/mL, 50.0 ng/mL, 60.0 ng/mL).

**Table S1.** Analytical performance parameters of the proposed method for determination of 3-AP in ALO and LIN drug substances.

| **Preparation** | **Initial 3-AP found**  **(ng/mL)** | **Added 3-AP**  **(ng/mL)** | **%Recovery*** | **%RSD** | **Regression equation** | **r^2^** |
| --- | --- | --- | --- | --- | --- | --- |
| ALO | 3.04 | 40.0  50.0  60.0 | 100  101  99.4 | | 4.57 | | --- | | 2.83 | | 2.60 | | y = 14.381x + 43.723 | 0.9998 |
| LIN | 1.45 | 40.0  50.0  60.0 | 97.3  101  100 | | 3.21 | | --- | | 3.46 | | 1.40 | | y = 16.133x + 23.333 | 0.9992 |

*Each result is the average of 3 different determinations.

**Table S2.** Analytical performance parameters of the proposed method for determination of 2-AP in PX and TX drug substances and dosage forms.

| **Preparation** | **Initial 2-AP found**  **(ng/mL)** | **Added 2-AP**  **(ng/mL)** | **%Recovery*** | **%RSD** | **Regression equation** | **r^2^** |
| --- | --- | --- | --- | --- | --- | --- |
| PX API | 1.68 | 40.0  50.0  60.0 | 101  100  99.4 | 2.77  2.06  2.69 | y = 36.842x + 61.835 | 0.9998 |
| TX API | 1.54 | 40.0  50.0  60.0 | 98.6  98.3  102 | 3.17  2.26  2.97 | y = 45.997x + 70.855 | 0.9989 |
| Brexin tablet (20 mg PX/tab.) | 20.7 | 40.0  50.0  60.0 | 98.3  100  100 | 2.18  2.64  3.67 | y = 43.588x + 903.02 | 0.9997 |
| Feldene ampoule (20 mg PX/amp.) | 0.77 | 40.0  50.0  60.0 | 99.3  101  99.3 | 0.17  1.57  1.35 | y = 36.854x + 28.482 | 0.9996 |
| Feldene capsule (20 mg PX/cap.) | 3.72 | 40.0  50.0  60.0 | 101  103  97.4 | 2.48  3.57  3.90 | y = 43.393x + 161.51 | 0.9973 |
| Dispercam suppository (20 mg PX/supp.) | 11.3 | 40.0  50.0  60.0 | 99.5  98.7  101 | 1.55  2.01  1.72 | y = 25.522x + 288.82 | 0.9995 |
| Feldene gel (0.5% PX gel.) | 2.08 | 40.0  50.0  60.0 | 99.7  99.3  101 | 1.62  1.56  2.51 | y = 39.647x + 82.586 | 0.9999 |
| Epicotil tablet (20 mg TX/tab.) | 1.67 | 40.0  50.0  60.0 | 98.6  102  99.3 | 2.26  0.78  2.81 | y = 44.092x + 73.566 | 0.9993 |
| Epicotil vial (20 mg TX/vial.) | 3.12 | 40.0  50.0  60.0 | 101  99.0  100 | 2.83  2.48  1.45 | y = 36.508x + 113.77 | 0.9997 |
| Soral capsule (20 mg TX/cap.) | Undetected | 40.0  50.0  60.0 | 96.7  102  100 | 1.49  3.43  3.64 | y = 40.298x + 7.9237 | 0.9987 |
| Epicotil suppository (20 mg PX/supp.) | 4.72 | 40.0  50.0  60.0 | 104  99.3  98.7 | 0.90  0.47  1.71 | y = 31.024x + 146.36 | 0.9983 |

*Each result is the average of 3 different determinations.
